# The Role of the Blood Neutrophil-to-Lymphocyte Ratio in Aneurysmal Subarachnoid Hemorrhage

**DOI:** 10.3389/fneur.2021.671098

**Published:** 2021-06-03

**Authors:** Lingxin Cai, Hanhai Zeng, Xiaoxiao Tan, Xinyan Wu, Cong Qian, Gao Chen

**Affiliations:** Department of Neurological Surgery, The Second Affiliated Hospital of Zhejiang University School of Medicine, Hangzhou, China

**Keywords:** aneurysmal subarachnoid hemorrhage, neutrophil to lymphocyte ratio, neuroinflammation, cerebrovascular disease, immune response, biomarkers

## Abstract

Aneurysmal subarachnoid hemorrhage (aSAH) is an important type of stroke with the highest rates of mortality and disability. Recent evidence indicates that neuroinflammation plays a critical role in both early brain injury and delayed neural deterioration after aSAH, contributing to unfavorable outcomes. The neutrophil-to-lymphocyte ratio (NLR) is a peripheral biomarker that conveys information about the inflammatory burden in terms of both innate and adaptive immunity. This review summarizes relevant studies that associate the NLR with aSAH to evaluate whether the NLR can predict outcomes and serve as an effective biomarker for clinical management. We found that increased NLR is valuable in predicting the clinical outcome of aSAH patients and is related to the risk of complications such as delayed cerebral ischemia (DCI) or rebleeding. Combined with other indicators, the NLR provides improved accuracy for predicting prognosis to stratify patients into different risk categories. The underlying pathophysiology is highlighted to identify new potential targets for neuroprotection and to develop novel therapeutic strategies.

## Introduction

Aneurysmal subarachnoid hemorrhage (aSAH) is the leading cause of death in stroke patients, with a mortality rate of ~40–50% (1, 2). More than 30% of survivors may develop severe disability and delayed neurological dysfunction due to complications such as delayed cerebral ischemia (DCI) and rebleeding (3). The progression of aSAH is individualized and rapid, so accurate risk stratification and prognosis prediction is challenging (4). A growing body of research has found that neuroinflammation and immune disorders after aSAH may play an important role in both early brain injury (EBI) and delayed neurological injury (5). Neutrophils and lymphocytes are vital inflammatory cells and participate in many pathogenic processes (6), such as endothelial injury, blood–brain barrier (BBB) destruction, microcirculation disturbance, and vasospasm (7). The dynamics of inflammatory cells and cytokines in the periphery can also contribute to systemic inflammatory response syndrome (SIRS) (8) and immunosuppression (9), which can perhaps increase the risk of infection (10).

The neutrophils-to-lymphocyte ratio (NLR) is a parameter that reflects inflammation and is regarded as a predictor of cardiovascular disease, pancreatitis, tumors, and other diseases (11). At present, several studies have shown that an increase in the NLR can predict adverse outcomes in patients with intracerebral hemorrhage (ICH) (12, 13). Because aSAH has a disease model similar to that of intracranial hemorrhage (14), the NLR may have the potential to assist in the prediction of the prognosis of aSAH.

The purpose of this review is to summarize existing evidence on the relationship between the NLR and aSAH and evaluate whether the NLR can be used to predict outcomes and as an effective biomarker for clinical management. We will also provide important insights into the underlying pathophysiological mechanisms and discuss the limitations of the current studies to make recommendations for future research.

## Pathophysiological Mechanism

### Neuroinflammation in aSAH

After aneurysm rupture, the blood enters the subarachnoid space and spreads to the brain through the cerebrospinal fluid (CSF). Erythrocytes degrade and release many bioactive and potentially toxic molecules (15), destroying blood vessels and initiating an inflammatory cascade reaction (16). Inflammation regulated by complex pathways plays a pivotal role in both neurological impairment and destruction (17). Microglia are resident immune cells in the central nervous system (CNS) and react rapidly to inflammation-associated neurological injuries (18). When activated by toll-like receptor 4 (TLR4), microglia switch to a pro-inflammatory phenotype (M1) and secrete a large number of pro-inflammatory chemokines and cytokines, such as interleukin-6 (IL-6), interleukin-1β (IL-1β) (19), tumor necrosis factor-alpha (TNF-α), and nitric oxide (NO) (20). These inflammatory factors can increase the expression of specific cell adhesion molecules (CAMs) on the luminal surface of endothelial cells (ECs), recruit macrophages and neutrophils to bind ECs, and enter the CNS (21). After migrating to the subarachnoid space, neutrophils are essential in clearing extravascular hemoglobin through the Hp–Hgb complex, and this process is a response to hemorrhage that promotes neural stability and recovery (22, 23).

The acute neuroinflammatory response influences delayed events such as rebleeding and DCI after aSAH (24, 25). The recruitment of neutrophils and lymphocytes damages the blood vessel wall, resulting in the thinning of the aneurysm wall and coagulation dysfunction (26, 27). Recent studies have highlighted the role of the neuroinflammation of the microvasculature in DCI (28). Inflammation disrupts the balance between endogenous vasodilators and vasoconstrictors, especially the production of NO and endothelin-1 (29). This imbalance can increase lipid peroxidation of the cell membrane and direct oxidative stress in smooth muscle cells (30), causing microperfusion disorders and cerebral ischemic infarction (31). The interaction of neutrophils with platelets and vascular endothelium has also been demonstrated (32). Some clinical evidence shows that the increase of neutrophils in CSF indicates vascular injury and may be an independent predictor of vasospasm, indicating the risk of DCI (33, 34). Further studies have found additional pathogenic mechanisms, such as EBI and apoptosis, destruction of the BBB (35), micro thromboembolism (36), and cortical diffuse depolarization (37), which are more or less linked to inflammation.

### Neutrophils and SIRS in aSAH

Neutrophils migrate to the damaged brain tissue at the earliest stage and reach a peak in the early stage (24–48 h) (38). Neutrophils interact with activated ECs and play an important role in aggravating the inflammatory response, leading to the destruction of the BBB, brain edema, hypoperfusion, and nerve cell injury (39). Targeting neutrophil function could mitigate cerebral hypoperfusion (40, 41), and inhibition of neutrophil–endothelial interactions markedly decreased neuronal cell death and reduced secondary brain injury (42). The mechanism pathway involves inflammatory factors released by neutrophils, such as reactive oxygen species, cathepsin (43), matrix metalloproteinase-9 (MMP-9), and myeloperoxidase (MPO) (44). Neutrophils show significant procoagulant effects and aggravate the disturbance of the microcirculation (45).

The activation of inflammation and an increase in neutrophils also occur in the peripheral immune system (46, 47). The interruption of the BBB or the CSF drainage system may serve as a communication channel for inflammatory factors (48, 49). The peripheral activation of innate immune cells after aSAH is regulated by the sympathetic nervous system (SNS) and the hypothalamic–pituitary–adrenal (HPA) axis (50, 51). These changes in the peripheral inflammatory status and the resulting symptoms may manifest as SIRS (52). The occurrence of SIRS with common characteristics, like body temperature, heart rate, blood pressure, leukocytosis, and platelet activation, usually leads to continuous tissue hypoperfusion and damage to microcirculatory blood flow (53, 54). In return, systemic hyperinflammation provoked by maladaptive innate immunity may activate innate immune signaling pathways in the CNS, increasing BBB destruction (55).

### Lymphocytes and Immunosuppression

Adaptive immunity is active in both the CSF and periphery after aSAH (56). Lymphocytes are found in the CNS after aSAH and are mainly involved in the protective mechanism against brain injury (57). Tregs play a key role in the elimination of inflammation and are the main brain-protective immunomodulator (58, 59). Tregs can reduce the inflammatory response by blocking the activation of the TLR4/nuclear factor-kappa B (NF-κB) signaling pathway, inducing the transformation of microglia toward a more favorable M2-like phenotype inhibiting the effect of neutrophil-derived MMP-9 to protect the integrity of the BBB and EC function (60, 61). The potent homeostatic function of Tregs in the peripheral immune system involves the inhibition of early immune overactivation that may result in an exhausted immune phenotype (62). In contrast, Th17 cells promote the inflammatory response (63) and are an important source of pro-inflammatory cytokines to aggravate brain injury (64).

A decrease in the total number of lymphocytes, especially Tregs, is found in aSAH patients (65). The profound loss of lymphocytes and the reduction of their activation are defined as pathophysiological immunosuppression, which is a long-lasting state that can last for several weeks and has a complex mechanism. The autonomic nervous system (ANS) and the SNS affect immune responses in the periphery and play a major role in immunosuppression (66). The stress response induced by injury activates the HPA axis and also leads to deficiency in the early activation of lymphocytes (67). Neutrophils release arginase 1 (ARG1), resulting in T-lymphocyte suppression in peripheral blood (68). Immunosuppression is also associated with changes in related cytokines released by lymphocytes, such as decreases in interferon-gamma (INF-γ) and interleukin-10 (IL-10) (69, 70). Lymphocytes are crucial for host defense against pathogens, and apparently, concomitant immunosuppression can increase vulnerability to systemic infection (71).

## Clinical Evidence

We reviewed relevant studies that associate NLR with aSAH to evaluate whether the NLR could serve as a biomarker to predict outcomes and be used as an effective biomarker for clinical management. A total of 11 studies assessed short- or long-term clinical outcomes according to the Modified Rankin Scale (mRS), early neurological deterioration, mortality, the occurrence of DCI, and other parameters. The synopses of the studies include the primary endpoint, inclusion criteria, and exclusion criteria, which are summarized in Table 1. The main characteristics of the patients enrolled in the studies are summarized in Table 2. The main conclusions are summarized in Table 3.

**Table 1:** Synopsis of the studies.

**References**	**Inclusion criteria**	**Exclusion criteria**	**Primary endpoint**
Jamali et al. (72)	Patients (≥18 years) with a diagnosis of SAH	Preexisting immunocompromised state, autoimmune disorders, hematologic disorders, and malignancies	Overall inpatient mortality; incidence of pneumonia during hospitalization
Wang et al. (73)	Patients with a diagnosis of SAH and rebleeding confirmed by CT. CTA or DSA was used to verify the presence of the intracranial aneurysm	The patients presented with other cerebrovascular diseases and intracranial tumors; multiple intracranial aneurysms; a malignant tumor; leukemia; hemolytic anemia; antiplatelet or anticoagulant-associated ICH; with pneumonia within 72 h following SAH	Rebleeding within 72 h following aSAH; 3 months outcome (favorable: mRS score 0–2; poor: mRS score 3–5)
Al-Mufti et al. (74)	Patients (≥18 years) with a diagnosis of SAH verified by admission CTA and DSA	SAH secondary to perimesencephalic bleeds, related to trauma, rupture of an arteriovenous malformation, or other causes	DCI (focal neurological impairment or a decrease in at least two points on GCS); poor outcome (death or moderate to severe disability: unable to walk or tend to bodily needs, mRS score of 4–6)
Giede-Jeppe et al. (75)	Patients with a diagnosis of SAH verified by admission CTA and DSA	Missing outcome data and missing NLR levels, patients suffering from infections	3 months outcome (favorable: mRS score 0–2; unfavorable: mRS score 3–6) and mortality; 12 months outcome and mortality; in-hospital complications
Ray et al. (76)	Patients with a diagnosis of SAH verified by admission CTA and DSA	Patients without appropriate CT scans, lost to follow-up or revoked consent; in-hospital death	1 year outcome (mRS score of 3–6, MoCA <26), DCI (a new hypodensity on CT and/or associated symptoms)
Wu et al. (77)	Patients with a diagnosis of SAH, verified by CT, received standard medical treatment	Patients with traumatic SAH, recent infectious diseases, and prior neurological conditions, including ischemic stroke, hemorrhagic stroke, or brain trauma	DCI (focal impairment or a decrease of at least 2 points on the GCS, without other causes)
Ogden et al. (78)	Patients (≥16 years) admitted to the emergency room and diagnosed with SAH on CT and then treated in the ICU	Patients whose data were incomplete, with head and general body trauma and with spontaneous intracerebral hematoma which drained into the ventricle	N/A
Yilmaz et al. (79)	Patients diagnosed as SAH by CT, MRI, and CTA	Patients with endocrinologic disorders, hematologic and rheumatologic diseases, malignancy, history of autoimmune diseases, immunosuppressive drug intake, acute or chronic infection, use of antiaggregant, anticoagulants, or analgesics	In-hospital mortality
Huang et al. (80)	Patients (≥15 years) with a diagnosis of SAH verified by ICD9^*^	Patients who have been previously admitted to ICU were excluded	Hospital death; 1 year mortality
Tao et al. (81)	Patients (≥18 years) with a first aSAH confirmed by DSA; received aneurysm repair treatment within 72 h after admission; initial blood sampling for laboratory test including NLR/PLR was limited within 24 h after ictus of hemorrhage	Patients with acute or chronic infection, history of autoimmune disease, previous stroke and recent cardiocerebrovascular disease, previous use of anticoagulant/antiplatelet medication, suffered aneurysm rebleeding before surgery and declined surgical intervention, other prior systemic diseases	DCI (a decrease of at least 2 points on the GCS) and 3 months outcome (poor: mRS score of ≥3)
Zhang et al. (82)	Patients (≥18 years) had an acute headache attack and underwent CT	Patients with acute or chronic inflammatory diseases, hematological diseases, cancers, autoimmune diseases, hepatic or renal insufficiency, cerebral hemorrhage, acute ischemic stroke or other trauma diseases, and patients whose clinical data were incomplete	Neutrophil, NLR, and PLR in SAH and non-traumatic acute headache

* *ICD9: code = 430 and sequence = 1 on MIMIC II database*.

*SAH, subarachnoid hemorrhage; aSAH, aneurysmal SAH; CT, computed tomography; CTA, CT angiography; DSA, digital subtraction angiography; ICH, intracerebral hemorrhage; mRS, Modified Rankin Scale; DCI, delayed cerebral ischemia; GCS, Glasgow Coma Scale; NLR, neutrophil-to-lymphocyte ratio; MoCA, Montreal cognitive assessment; ICU, intensive care unit; PLR, platelet-to-lymphocyte ratio*.

**Table 2:** Characteristics of the included patients.

**References**	**Patients,** ** number**	**Age, years**	**Female, %**	**Clinical severity**	**NLR values**	**Time onset to sample**
^***^Jamali et al. (72)	44	52 ± 12 vs. 59 ± 19	21 (66%) vs. 6 (50%)	GCS: 13 ± 3 vs. 5 ± 4; FG: 3.59 ± 0.67 vs. 3.75 ± 0.62	11.53 vs. 17.85	<24 h
^**^Wang et al. (73)	716	52.23 ± 12.74 vs. 54.99 ± 11.59	14 (46%) vs. 437 (64%)	HHG > 3, 8 (26.7%) vs. 107 (15.6%); FG > 2, 22 (73.3%) vs. 287 (41.8%)	11.27 (6.31–16.19) vs. 5.50 (2.71–10.64)	16 (7.25–20) vs. 14 (8.0–17)
Al-Mufti et al. (74)	1,067	Age > 53, 589 (55%)	731 (69%)	HHG ≥ 3, 645 (61%); GCS <8, 301 (29%)	NLR ≥ 5.9, 768 (72%)	<24 h
^*^Giede-Jeppe et al. (75)	319	51 (43–59) vs. 57 (49–70)	104 (65.8%) vs. 117 (72.7%)	HHS: 2 (1–3) vs. 4 (2–5); GCS: 15 (13–15) vs. 9 (3–13); WFNS: 1 (1–2) vs. 4 (2–5)	5.8 (3.0–10.0) vs. 8.3 (4.5–12.6)	Admission
Ray et al. (76)	44	54.7 ± 12.8	31 (70.5%)	HHG > 3, 8 (28.2%); FG > 2, 29 (65.9%); WFNS > 3, 17 (38.6%)	^#^-0.78 (−1.42, −0.13) vs. −1.13 (−1.47, −0.78)	N/A
Wu et al. (77)	122	55.3 ± 10.6	48 (39.3%)	HHS > 3, 4 (19.7%); FG > 2, 109 (89.3%)	10.7 ± 8.2	<72 h
Ogden et al. (78)	54	58.50 ± 16.52	24 (44.4%)	^##^GSC: 15 ± 3.64 vs. 12 ± 3.41 vs. 10 ± 5.09; FG: 3 ± 0.79 vs. 4 ± 0.78 vs. 2 ± 0.63	^##^7.71 ± 13.49 vs. 11.07 ± 7.86 vs. 2.51 ± 8.71	N/A
Yilmaz et al. (79)	152	52.94 ± 17.04	94 (61.8%)	^###^FG: 4 (1–4) vs. 3 (1–4)	^###^8.48 (0.1–54.8) vs. 3.56 (0.46–32.9)	<24 h
Huang et al. (80)	274	59 ± 16	164 (59.9%)	SAPS: 12.5 ± 5.6	8.7 ± 9.2	N/A
Zhang et al. (82)	54	56.91 ± 13.45	38 (70.4%)	N/A	9.88 ± 7.68	<24 h

* *3 months mRS score: 0–2 (n = 158) vs. 3–6 (n = 161)*.

** *Rebleed group (n = 30) vs. non-rebleed group (n = 686)*.

*** *Alive group (n = 32) vs. dead group (n = 12);*

# *DCI (n = 27) vs. no DCI (n = 13). Data show 95% confidence interval*.

## *Patients with angiography of negative spontaneous (n = 20) vs. patients with SAH originating from anterior communicating artery aneurysm (n = 18) vs. patients with traumatic SAH (n = 16)*.

### *Aneurysmal (n = 99) vs. non-aneurysmal SAH (n = 53)*.

*Data are mean or mean ± standard deviation, median (interquartile range), and number of patients (%), unless otherwise indicated*.

*NLR, neutrophil-to-lymphocyte ratio; GCS, Glasgow Coma Scale; FG, Fisher grade; HHG, Hunt–Hess grade; WFNS, World Federation of Neurosurgical Societies grade; SAPS, simplified acute physiology score; DCI, delayed cerebral ischemia; SAH, subarachnoid hemorrhage*.

**Table 3:** Synthesis of the main findings.

**References**	**Main findings**
Jamali et al. (72)	NLR ≥ 12.5 at admission predicts higher inpatient mortality in patients with aSAH.
Wang et al. (73)	Higher NLR predicts the occurrence of rebleeding and poor outcome at 3 months, and NLR combined with Fisher grade significantly improves the prediction of rebleeding following aSAH. The AUC values of the NLR and combined NLR–Fisher grade model were 0.702 and 0.744 (sensitivity was 39.94%, and specificity was 100%), respectively, for predicting rebleeding. After PSM, the optimal cutoff value for NLR as a predictor for rebleeding following aSAH was determined as 5.4 (sensitivity was 83.33%, and the specificity was 63.33%).
Al-Mufti et al. (74)	Admission NLR predicted development of DCI, a sensitivity of 63% and specificity of 53% for predicting DCI.
Giede-Jeppe et al. (75)	NLR represents an independent parameter associated with unfavorable functional outcome for aSAH; an NLR of 7.05 was identified as the best cutoff value to discriminate between favorable and unfavorable outcomes.
Ray et al. (76)	NLR trends showed a significant initial decline among those without DCI, a gradual rise in those developing DCI, and an early immune-depressed state after aSAH.
Wu et al. (77)	NLR may be a practical predictor for the occurrence of DCI in SAH patients. NLR possessed the largest AUC. The best cutoff value of NLR was 11.47. The sensitivity and specificity of NLR were 58.1 and 82.3%, respectively, for predicting DCI.
Ogden et al. (78)	NLR could be predictive for etiological factors (traumatic SAH or spontaneous SAH) of patients who were admitted unconscious to the emergency room with SAH detected on radiological imaging. An NLR <4.17 was 81% sensitive and 75% specific in discriminating traumatic SAH patients from angiography-negative SAH patients, indicating that this hemorrhage might be traumatic; An NLR <3.62 was 81.3% sensitive and 83.3% specific in discriminating traumatic SAH patients from aneurysmal SAH patients, indicating that this hemorrhage might be traumatic
Yilmaz et al. (79)	Higher NLR values were significantly related to mortality rates.
Huang et al. (80)	NLRs were significantly associated with hospital death, and higher 1 year mortality
Tao et al. (81)	NLR is independently related to DCI and functional outcome at 3 months after aneurysm repair. The combination of NLR and PLR showed a better predictive value than each alone for DCI and poor outcome
Zhang et al. (82)	NLR was a useful and potential tool in distinguishing between SAH and non-traumatic acute headache. The best cutoff value of NLR was 4. The sensitivity and specificity of NLR were 98.59 and 64.81%, respectively, for distinguishing patients with SAH from those with non-traumatic acute headache.

*NLR, neutrophil-to-lymphocyte ratio; aSAH, aneurysmal subarachnoid hemorrhage; AUC, area under curve; PSM, propensity score matching; DCI, delayed cerebral ischemia*.

### Prediction of Adverse Clinical Outcomes

The relationship between the NLR and unfavorable clinical outcomes was investigated in a retrospective cohort study. Giede-Jeppe et al. described 319 patients in a tertiary inpatient center in Germany and recorded the mRS score at 3 and 12 months. It was found that the NLR value of patients with an unfavorable prognosis was significantly increased. After adjustment, the NLR remained an important factor in predicting unfavorable outcomes in patients. Through receiver operating characteristic (ROC) analysis, an NLR ≥7.05 was determined as the best cutoff value to predict unfavorable outcomes, which indicated an mRS score of 3–6 after 3 months (75). Wang et al. also found that there was a positive correlation between the NLR and the Hunt–Hess grade and that the NLR could predict the adverse outcomes of patients with an mRS score of 3–5 after 3 months (73). In a study that included 247 patients, the NLR was proven to be an independent predictor of adverse outcomes after 3 months, with a sensitivity of 74.5% and a specificity of 69.3% (81).

The NLR was also an independent marker of mortality in patients with aSAH. Huang et al. found that the increase in the NLR in intensive care unit (ICU) patients was significantly associated with hospital mortality and 1-year mortality (80). Jamali et al. found that the NLR value of patients who died in the hospital was significantly higher than that of surviving patients, and the in-hospital mortality rate of patients with an NLR ≥12.5 increased significantly. In this study, the effects of the absolute neutrophil count and absolute lymphocyte count on mortality were compared separately, and the results showed that there was no difference (72). Yilmaz et al. also found that the NLR was associated with the Fisher score and mortality and was a simple indicator of aSAH patient severity and short-term mortality (79).

### Prediction of the Risk of Complications

The main complications of aSAH are DCI and rebleeding, which are common clinically and cause severe disability and delayed neurological dysfunction. A single-center, prospective, observational cohort study of 1,067 patients at Columbia University Medical Center demonstrated that the NLR on admission could predict the occurrence of DCI. They found that in the multivariate model, an increased NLR was associated with a poor Hess grade on admission, Caucasian race, the location of the anterior aneurysm, loss of consciousness at onset, and an increased bleeding volume (Fisher ≥ 3). After controlling for known predictors such as age, poor clinical grade on admission, bleeding volume, and mean arterial pressure on admission, the admission NLR could predict the occurrence of DCI, and an NLR ≥5.9 on admission predicted a 2-fold increase in the risk of DCI (74).

Wu et al. also found that the NLR may be a practical index to predict the occurrence of DCI in aSAH patients. They studied 122 patients, and 43 of them developed DCI during hospitalization and had an increased white blood cell count, neutrophil count, and NLR and a lower lymphocyte count. The NLR value was the most predictive variable among the four variables, and the best predictive value was 11.47. The sensitivity and specificity were 58.1 and 82.3%, respectively (77). The findings of Tao et al. also supported these results, as they found that an increased NLR was an independent factor that predicted the occurrence of DCI, with a sensitivity of 87.3% and a specificity of 48.4% (81). Ray et al. carried out further studies on the dynamics of the NLR and found that NLR trends showed a significant initial decline followed by a gradual rise among those without DCI, whereas the NLR was persistently low in those patients that developed DCI in an early immune-depressed state after aSAH (76).

The relationship between the NLR and rebleeding was proven as well. In a study that included 716 patients with aSAH, rebleeding occurred in 4.19% of patients. Univariate analysis showed that the NLR in patients with rebleeding was significantly higher than that in patients without rebleeding. In the multivariate analysis, the NLR and Fisher grade were risk factors for rebleeding. An increased NLR predicted the occurrence of rebleeding and poor prognosis after aSAH. In addition, a model combining the NLR and the Fisher grade was more valuable for predicting rebleeding than a single NLR or Fisher rating model, with a specificity of 100% (73).

## Practice Prospects

### Prediction of Outcomes

The mortality rate of aSAH is quite high, and survivors are typically left with prolonged functional impairments, but accurate estimation of the prognosis of aSAH patients has been challenging (83). At present, the most important prognostic determinants for the aSAH outcome are the neurological grade on initial examination, radiographic results, and age (84, 85). However, subjective differences are inevitable in using the grading scales (86), and the sensitivity of the radiograph depends on the time interval between symptom occurrence and image acquisition (87). Researchers are hard-pressed to find new, feasible, and accurate evaluation and prediction methods.

Severe subarachnoid hemorrhage is associated with the strong early inflammatory response (88), and the most direct and easily observed result is the change in peripheral inflammatory biomarkers, such as the neutrophil and lymphocyte counts (89, 90). In general, an increase in neutrophils in peripheral blood is a manifestation of inflammation in the CNS and may be related to the development of extracerebral organ immune dysfunction. And SIRS associated with neutrophils is also related to poor clinical grade and independently predicts poor outcomes (91). A decrease in lymphocytes suggests the loss of neuroprotection and the impairment of native immunity with immunosuppression in the periphery (92). Therefore, NLR is a peripheral biomarker that conveys information about the inflammatory burden in terms of both innate and adaptive immunity and may fill the current lack of prognosis prediction.

It is understood that NLR is easy to utilize clinically without additional costs. As a ratio, the NLR remains more reliable and stable than a single blood parameter and is not affected by factors such as dehydration, overhydration, and blood sample treatment (93, 94). In addition, the NLR is more specific, multidimensional, and sensitive when slight changes in the inflammatory status of patients are difficult to detect (95). In conjunction with imaging results and the neurological grade, the NLR is of great value for the risk stratification process and the guidance of early prevention (96).

### Prediction of Rebleeding, DCI, and Infection

The complications of aSAH include rebleeding, DCI, hydrocephalus, increased intracranial pressure, and seizures (97). Previous studies have highlighted rebleeding and DCI, which contribute to high rates of disability and neurological dysfunction in survivors and are independent predictors of unfavorable outcomes (98). Rebleeding events are defined as a new hemorrhage apparent on repeat computed tomography (CT) with or without new symptoms and are viewed as the cerebrovascular equivalent of a second hit (99, 100). One of the causes of adverse clinical outcomes is the delayed diagnosis and treatment of rebleeding (101, 102). There is no sensitive and facile prediction method for prioritizing early treatment, such as early or ultra-early aneurysm repair and the use of antifibrinolytic drugs (103, 104). The NLR has been shown to predict the occurrence of rebleeding, which may be explained by the mechanism that neutrophil recruitment changes the stability of the ruptured aneurysm wall (73, 105). The present study on the NLR and rebleeding is limited but indicates potential predictors and new strategies for addressing neuroinflammation in rebleeding.

DCI generally occurs within 2 weeks after subarachnoid hemorrhage and is caused by cerebral perfusion defects (106), resulting in the deterioration of neurological function and a dramatically decreased quality of life in most survivors (107). The challenge is how to predict the occurrence of DCI and then formulate effective preventive strategies to reverse the development of DCI in advance (108). At present, four clinical studies have proven that the NLR is an independent prognostic factor for the occurrence of DCI with strong sensitivity and specificity (74, 76, 77, 81). This implies that the NLR is promising as a new predictor for DCI to help classify patients and direct preventive treatment to those who are most likely to benefit.

Increasing evidence shows that immune system disorders after aSAH and the risk of infection are increased (109). Immunosuppression induced by a significant decrease in peripheral lymphocytes is perhaps the vital reason for the increased risk of infection, especially in critically ill patients (110). Patients with SIRS also tend to become infected (111). Within 24 h after aSAH, an increase in the NLR may indicate the occurrence of intense early inflammation, which could develop into a strong late inflammatory response (112). Patients with a high NLR are more likely to develop pneumonia during hospitalization (113), and an NLR ≥5.9 is associated with pulmonary edema and high fever (74). The average number of neutrophils in patients with pneumonia is significantly increased (72). In particular, patients with DCI have a higher NLR and are more likely to be infected at a later stage (76). The NLR is an accessible indicator to evaluate immunity status and infection risk and can help adjust treatment strategies for preventing postoperative infectious complications (114).

### Other Roles of the NLR and Implications for Future Research

Further study could focus on the comparison of the role of neutrophils and lymphocytes in different neurological diseases. Current studies have shown that NLR is associated with a variety of diseases, such as ischemic stroke, other ICH (115), and traumatic brain injury. A number of studies have shown that NLR was predictive of short-term functional outcome in acute ischemic stroke (AIS) (116). A high NLR was also found to be an independent predictor for the complications following AIS including cerebral edema (117), hemorrhagic transformation after ischemia (118), and the development of ICH after endovascular treatment (119).

Neuroinflammation is prevalent in these conditions and plays with similar mechanisms. In the early stage of both SAH and AIS, the activation of microglia by neutrophil is vital for following cascade of inflammation, with similar inflammatory mediators and pathways (120). Moreover, Tregs also play a major role in immunomodulatory function and neuroprotection after AIS (121). Valuable findings and breakthroughs may occur through comparing the similarities and differences of pathophysiological mechanisms about neuroinflammation among these medical conditions.

Furthermore, systematic and quantitative analyses of relevant clinical studies are needed to explore different levels and trends of NLR in multiple diseases, which could develop potential and guiding clinical applications. Ogden et al. studied patients in the neonatal intensive care unit (NICU) and found that the NLR was higher in aSAH than in traumatic subarachnoid hemorrhage (78). Eryigit et al. also found that the NLR can be used in distinguishing aSAH and migraine, with different levels and changes of NLR (122). The different cutoff values may be useful in specific classifications for etiologies.

NLR is proven to be a useful and potential tool in distinguishing between aSAH and SAH of other etiologies (82). The potential mechanism may be that neuroinflammation plays a significant role during the formation and rupture of cerebral aneurysms (123). Neutrophil infiltration and pro-inflammatory cytokine overexpression cause thinning of the aneurysm wall and coagulation dysfunction (124, 125). However, the hypothesis that the NLR may be used as an early warning index before aneurysm rupture requires more exploration regarding the pathology of aneurysm progression and neuroinflammation.

It is encouraging that combining the NLR with other indicators can enhance the predictive value. The combined NLR–Fisher score model significantly improves the ability to predict the outcome of aSAH with higher sensitivity and specificity (74). Combining the NLR with the platelet-to-lymphocyte ratio (PLR) is more sensitive for aSAH than either indicator alone (81, 126). The prognosis of neurological diseases is affected by a number of factors, and combining NLR with other potential indicators may be a potential way to improve the prediction accuracy and clinical value of NLR. Lattanzi et al. found that the accuracy of outcome prediction in acute ICH would increase when NLR combined with the modified ICH score (127). NLR combined with patient characteristics like gender, a history of hypertension, diabetes mellitus, and smoking could present high diagnostic accuracy in ischemic stroke (128, 129). NLR combined with PLR was also found to be more meaningful in the early clinical detection of post-stroke depression than either alone (130).

Further studies may focus on the application of the NLR as a new potential target for developing novel therapeutic strategies (131). Neutrophils and lymphocytes show the potential to have substantial beneficial effects. Preclinical studies have shown that selective limitation of neutrophil activity can reduce neuroinflammation and neuronal apoptosis, eventually protecting the microcirculation and improving prognosis (132). Therapeutic strategies for T lymphocytes, such as the use of cyclosporine, have also been shown to be effective in some studies (133, 134). Many studies have evaluated drugs aimed at inhibiting inflammation, but the results have been mixed and not as obvious as originally hoped (135). It is not advisable to inhibit overall inflammation, and immunomodulatory methods should aim to block harmful effects selectively rather than functioning as an extensive, non-selective anti-inflammatory therapy (136, 137). Future treatment strategies should focus on subtle regulation of the immune response, which requires the precise assessment of inflammation, and the NLR may be a promising index for this.

## Limitation

There are many areas of uncertainty regarding the evaluation of the NLR as a prognostic marker in aSAH and new therapeutic targets. To some extent, the conclusions of clinical studies may overestimate the causality and clinical correlation between the NLR and aSAH, for the existence of confounding factors is inevitable (138). In addition, the admission criteria of studies are not harmonized or standardized, and some are not strict enough. Moreover, the sampling time of all studies is at the time of admission, which ignores the fact that the NLR is a dynamic index (23, 139). The current research can only prove the predictive role of the NLR on admission; a further prospective study is needed to provide time trend data to explore the overall dynamic changes.

Undoubtedly, further multicenter, large-sample, prospective, standardized evaluation studies are needed to confirm the detailed mechanisms and causal link between the NLR and aSAH. Additionally, all candidate biomarkers and molecules in the upstream pathways should be further explored for the detection of target antigens that link central and peripheral inflammation and to help identify other new targets (140).

## Conclusions

Present studies have demonstrated that NLR may be useful for predicting clinical outcome and the risk of complications in patients with aSAH. And the underlying pathophysiology mechanism of neuroinflammation is highlighted to further support this conclusion. However, NLR may present an insufficient specificity when it plays a predictive role in a variety of medical conditions. Combining NLR with other relevant prognosis factors may be a potential way to improve the predictive ability and clinical value of NLR. Despite the complexities of neuroinflammation and deficiency of present studies, modulating levels of neutrophils and lymphocytes and relevant signal transduction pathways could be a clinical therapeutic intervention in aSAH.

## Author Contributions

LC and HZ wrote and revised the manuscript. XW and XT helped in finding references. All authors contributed to the article and approved the submitted version.

## Conflict of Interest

The authors declare that the research was conducted in the absence of any commercial or financial relationships that could be construed as a potential conflict of interest.

## Abbreviations

aSAH: aneurysmal subarachnoid hemorrhage
NLR: neutrophil-to-lymphocyte ratio
EBI: early brain injury
DCI: delayed cerebral ischemia
BBB: blood–brain barrier
SIRS: systemic inflammatory response syndrome
CSF: cerebrospinal fluid
CNS: central nervous system
TLR4: toll-like receptor 4
IL-6: interleukin-6
IL-1β: interleukin-1β
TNF-α: tumor necrosis factor-alpha
NO: nitric oxide
CAMs: cell adhesion molecules
MMP-9: matrix metalloproteinase-9
PSGL-1: P-selectin glycoprotein ligand-1
MPO: myeloperoxidase
mRS: Modified Rankin Scale
ANS: autonomic nervous system
SNS: sympathetic nervous system
HPA: hypothalamic–pituitary–adrenal.
